# Successful Diagnosis of Sengers Syndrome Using a Comprehensive Genomic Analysis

**DOI:** 10.1002/mgg3.70048

**Published:** 2025-01-16

**Authors:** Kohta Nakamura, Yukiko Yatsuka, Sachie Naito, Akira Hasegawa, Takeya Kasukawa, Atsushi Kondo, Yoshihito Kishita, Yohei Sugiyama, Takanori Onuki, Tomohiro Ebihara, Tomoko Tsuruoka, Takuya Fushimi, Akira Ohtake, Kei Murayama, Atsuko Imai‐Okazaki, Yasushi Okazaki

**Affiliations:** ^1^ Diagnostics and Therapeutics of Intractable Diseases, Intractable Disease Research Center, Graduate School of Medicine Juntendo University Tokyo Japan; ^2^ Department of Pediatrics Funabashi Central Hospital Chiba Japan; ^3^ Laboratory for Large‐Scale Biomedical Data Technology RIKEN Center for Integrative Medical Sciences Kanagawa Japan; ^4^ Department of Life Science, Faculty of Science and Engineering Kindai University Osaka Japan; ^5^ Department of Pediatrics, Faculty of Medicine Juntendo University Tokyo Japan; ^6^ Department of Metabolism Chiba Children's Hospital Chiba Japan; ^7^ Department of Neonatology Chiba Children's Hospital Chiba Japan; ^8^ Department of Pediatrics & Clinical Genomics Saitama Medical University Saitama Japan; ^9^ Center for Intractable Diseases Saitama Medical University Hospital Saitama Japan; ^10^ Laboratory for Comprehensive Genomic Analysis RIKEN Center for Integrative Medical Sciences Kanagawa Japan

**Keywords:** genetic analysis, mitochondrial disease, mitochondrial DNA depletion syndrome, mitochondrial respiratory chain complex deficiencies, Sengers syndrome

## Abstract

**Background:**

Sengers syndrome is an autosomal recessive mitochondrial DNA depletion syndrome characterized by hypertrophic cardiomyopathy, congenital cataracts, skeletal myopathy, exercise intolerance, and lactic acidosis. Dysfunction of acylglycerol kinase (*AGK*) is responsible for the disease, and several *AGK* gene variants have been reported.

**Methods:**

We employed a comprehensive genomic analysis approach, including whole‐genome sequencing and RNA sequencing, combined with various bioinformatics tools.

**Results:**

Our analysis successfully diagnosed Sengers syndrome in a patient by detecting a known pathogenic variant and a previously unreported large deletion involving the *AGK* gene in a segmental duplication.

**Conclusion:**

This study demonstrates the effectiveness of combining multiple genomic analysis approaches for the accurate diagnosis of Sengers syndrome, particularly in cases involving complex genetic variations such as large deletions in segmental duplications.

## Graphical abstract

Sengers syndrome, characterized by hypertrophic cardiomyopathy, congenital cataracts, skeletal myopathy, exercise intolerance, and lactic acidosis, is caused by mutations in the *AGK* gene. This study reports the successful diagnosis of Sengers syndrome using comprehensive genomic analysis, identifying both a known pathogenic *AGK* variant and a previously unreported large deletion in a segmental duplication region.

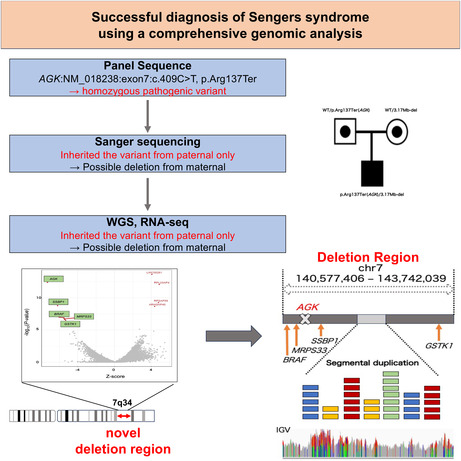

## 1 Introduction

Sengers syndrome is an autosomal recessive mitochondrial DNA depletion syndrome. First described in 1975, the syndrome is characterized by hypertrophic cardiomyopathy, congenital cataracts, skeletal myopathy, exercise intolerance, and lactic acidosis (Sengers et al. 1975). Based on these distinct phenotypes, Sengers syndrome is a clinically recognizable mitochondrial disease (Haghighi et al. 2014). The gene responsible for Sengers syndrome is acylglycerol kinase (*AGK*), the loss of which results in a decreased adenine nucleotide translocator in the inner mitochondrial membrane (Mayr et al. 2012). Various *AGK* variants have been reported, including missense variants, splicing variants, and short indels (Barbosa‐Gouveia et al. 2021; Wu et al. 2023). Here, we report the successful diagnosis of Sengers syndrome with a known pathogenic variant and a large deletion including the *AGK* gene within a segmental duplication by combining different genomic analysis approaches. Our comprehensive genomic approaches identified chromosomal deletions in segmental duplication regions, which may be applicable to identify novel chromosomal deletions in complex regions causative for other diseases.

## 2 Methods

### 2.1 Case Presentation

This male patient was born weighing 2.3 kg at 39 weeks of gestation (Apgar score 10/10). The patient was referred to Funabashi Central Hospital at 3 days after birth because of intermittent hypoxia and apnea after sucking. In addition to tachypnea and hypoxia, the examination after admission revealed elevated serum lactic acid (8.74 mmol/L) and cardiac hypertrophy. The patient was treated with a mitochondrial drug cocktail consisting of vitamin B1, B2, B12, vitamin C, biotin, vitamin E, coenzyme Q10, and L‐carnitine.

Congenital cataract was observed at 2 months of age. Although cardiac hypertrophy was improved during the follow‐up period, echocardiography performed at 154 days after birth revealed cardiac dilatation (left ventricular end‐diastolic diameter: LVIDd 29 mm, *Z*‐score 3.5) with reduced left ventricular systolic function (fractional shortening: FS 18%). Despite intensive care, the patient died of circulatory failure at 158 days of age.

Skin fibroblasts obtained from the patient were sent to Chiba Children's Hospital for evaluation of suspected mitochondrial diseases. Written informed consent was obtained from the patient's parents. Approval for enzymatic and genetic analyses was obtained from the appropriate ethics review boards at each institution. Echocardiographic images are shown in Data S1

### 2.2 Mitochondrial Respiratory Chain Complex Activities

The activities of mitochondrial respiratory chain (MRC) complexes I, III, and IV were measured using mitochondria obtained from cultured skin fibroblasts or the crude supernatant after centrifugation at 600 × *g* from liver and heart tissues, obtained from autopsies. Enzyme activity of each complex was estimated as a percentage of the normal control mean relative to citrate synthase (CS) activity. Thresholds for a decrease in enzyme activity were < 40% in a cell line or < 30% in a tissue, as previously reported (Bernier et al. 2002; Kirby et al. 2007).

### 2.3 Genetic Analysis

We performed several genetic analyses including custom panel sequencing, whole genome, sequencing, RNA sequencing combined with various bioinformatics tools. Detailed protocols are summarized in Data S2. We performed the PCR amplification experiment using trio gDNA to confirm whether the suspected large deletion was maternally derived or de novo. The PCR primer set was designed outside the deletion region (primer 1: 5'‐CCTTTGCATTGTGCTTTTCA‐3′, primer 2: 5'‐AAGGCGCTCTCTCAATTCC‐3′), and the extension time for each cycle was 1 min. The PCR target region spans 3.17 Mb, so the PCR will not work if there is no deletion. Otherwise, if a deletion is present, a PCR amplification product of approximately 1 kb will be produced.

## 3 Results

**FIGURE 1:**
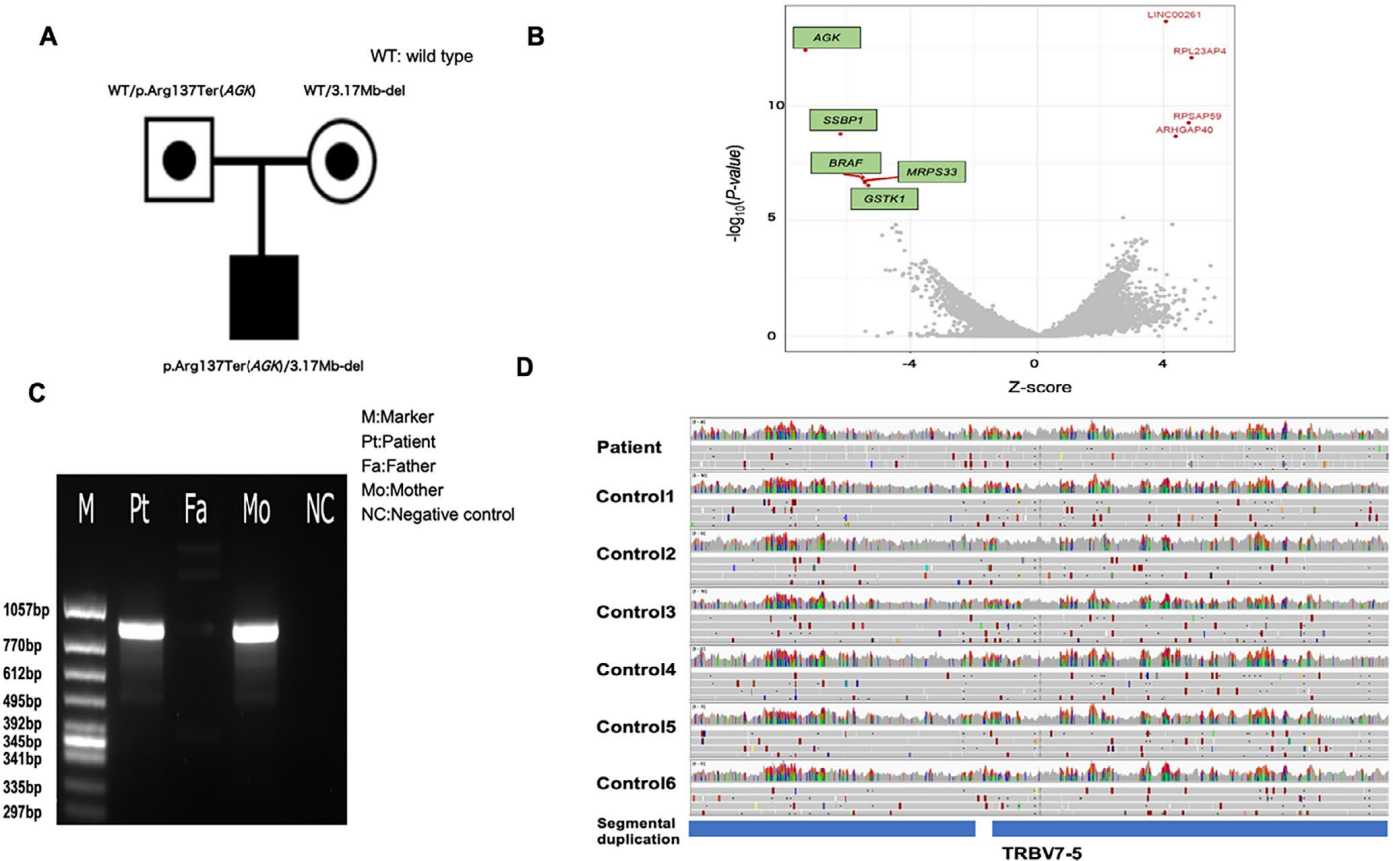
Pedigree, identified variants, and RNA‐seq results. (A) Patient pedigree. (B) Analysis of RNA‐seq data for patient's skin fibroblasts from the patient using OUTRIDER. Green indicates genes with decreased expression. The horizontal axis shows the *Z*‐score (positive values indicate increased expression and negative values indicate decreased expression). The vertical axis indicates −log10(*p*‐value). (C) Result of deletion detecting PCR. M: Marker, Pt: Patient, Fa: Father, Mo: Mother, NC: Negative Control (D) Visualization of segmental duplications in HDR case and control groups. Segmental duplications were detected in both cases and controls using IGV Genome browser. Segmental duplication files were downloaded from NCBI.

Mitochondrial Respiratory Chain (MRC) enzyme activity analyses revealed severe complex I defects in the heart (4.8%), while skin fibroblasts and liver activity levels were not different from normal control (120.9% and 50.8%, respectively) (Data S3). Mitochondrial DNA copy‐number analysis showed no significant mitochondrial depletion in the patient's heart or liver. Panel sequencing identified a known homozygous pathogenic variant in *AGK* (NM_028238.4 exon7, c.409C>T, p.Arg137Ter) (Mayr et al. 2012). This variant has already been reported as pathogenic in ClinVar. Familial segregation analysis by Sanger sequencing revealed a paternal heterozygous variant with no maternal inheritance (Figure 1A). We suspected that there was a large chromosomal deletion containing *AGK* that was maternally inherited or de novo arising; therefore, we performed WGS and RNA‐seq (Omichi et al. 2023). RNA‐seq analysis revealed decreased expression not only of *AGK* but also of four neighboring genes, *SSBP1*, *BRAF*, *MRPS33*, and *GSTK1* (Figure 1B) (Brechtmann et al. 2018). Among five bioinformatics pipelines with default parameters, only Estimation by Read Depth with Single‐nucleotide variants (ERDS) identified a candidate deletion of approximately 3 Mbp (chr7:140,481,601–143,650,800) in close proximity to the five genes with decreased expression levels. Three bioinformatics pipelines identified different candidate deletions: HDR‐del detected two separate regions, chr7:140,577,406–142,453,641 and chr7:142,501,509–143,742,039, Copy Number Variation nator (CNVnator) identified regions of approximately 1.4 Mbp at the start edge of the *AGK* gene, and Autozygosity Mapper (AutoMap) detected two separate regions at the start and end of the gene (chr7:140,429,030–142,467,484 and chr7:142,524,657–143,537,212). Using Manta and LUMPY, we did not detect any candidate deletions surrounding the *AGK* gene. Direct Polymerase Chain Reaction (PCR) sequencing using Trio samples revealed a deleted region at chr7:140,481,623–143,646,987, close to the regions detected by ERDS, inherited from the patient's mother (Figure 1C). Considering the inconsistencies between the results obtained by different bioinformatics pipelines, we suspected that the deletion is in a region with many segmental duplications, as shown in Figure 1D.

### 3.1 Improved HDR‐del Algorithm for the Accurate Identification of a Deletion Within a Segmental Duplication

**FIGURE 2:**
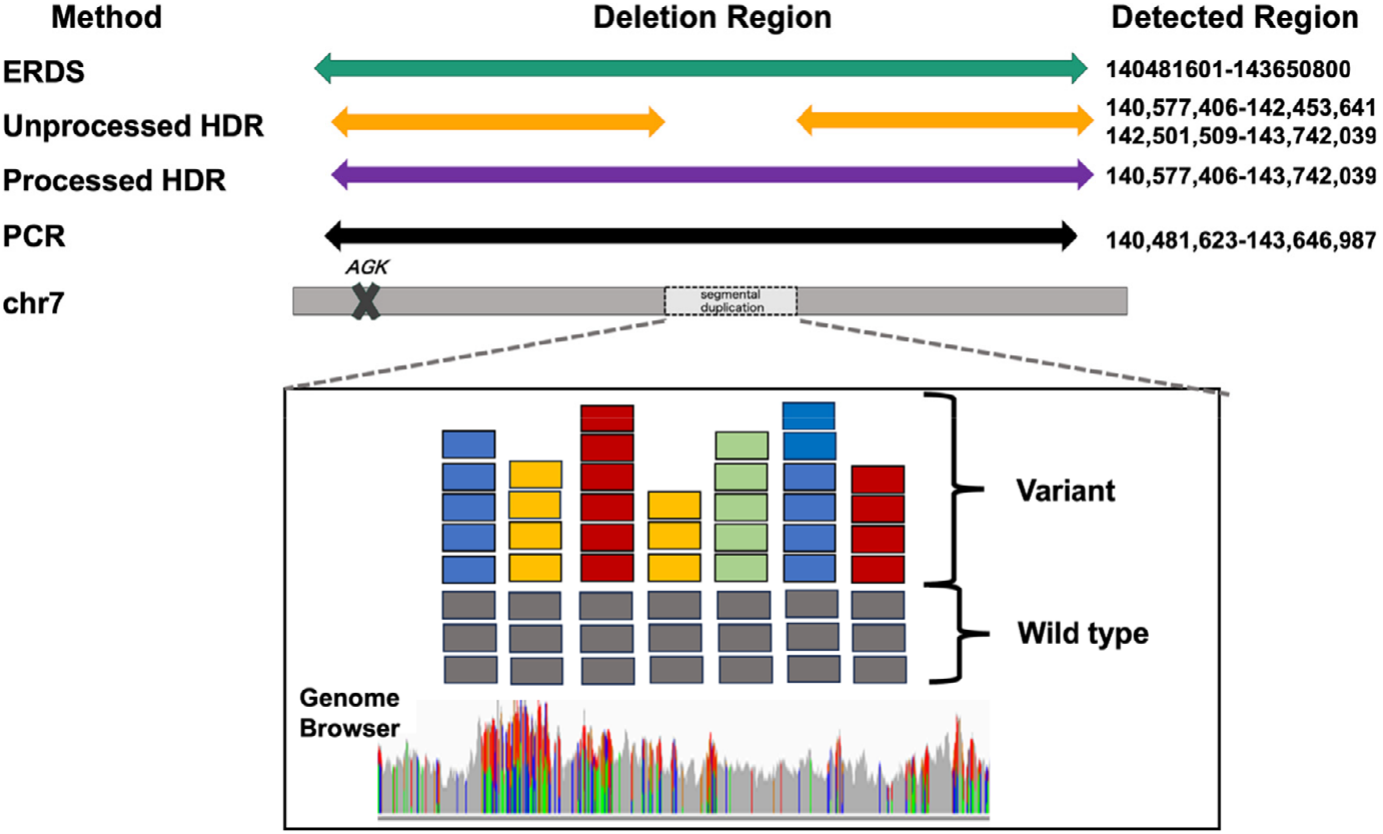
Comparison of HDR‐del results with those of other methods Comparison of the results obtained using ERDS, the most accurate bioinformatics tool available, and unprocessed HDR‐del, processed HDR‐del, and PCR methods. Regions where a segmental duplication was confirmed are indicated by angle brackets.

The HDR‐del method only detected simple deletions, excluding regions with segmental duplications or gene rearrangements (Imai‐Okazaki et al. 2017) (Imai et al. 2015). For deletions in complex regions, as in our case, this method is not suitable. Indeed, the HDR‐del method revealed two regions separated by segmental duplications in Data S4. We used the IGV genome browser to evaluate the undetected region. To improve the accuracy of HDR‐del, we first visualized the genotypes located in undetectable regions. We identified a 4.7 kb region that was not detected by HDR‐del and contained many heterozygous variants not only in the patients but also in the control group (Figure 1D). All the heterozygous genotypes in the undetectable regions are visualized in Data S5. Using the National Center for Biotechnology Information (NCBI) database (Krusche et al. 2019), we found that this region that was not detected by HDR‐del was within a reported segmental duplication. We removed segmental duplications registered in the NCBI database from the input file for HDR‐del. Using HDR‐del with processed data, the deletion at chr7:140,577,406–143,742,039 was successfully identified, consistent with the regions detected by PCR and ERDS (Table 1). Deletion regions identified by ERDS, the most accurate bioinformatics tool available, as well as unprocessed HDR‐del, Processed HDR‐del, and direct PCR sequencing are compared in Figure 2 and Data S6.

**TABLE 1:** Processed HDR‐del results.

*p*‐value	Genome	Annotation
0.04	chr7:140,577,406–143,742,039	*RAB19*, *MKRN1*, *DENND2A*, *Y_RNA*, *RN7SL771P*, *ADCK2*, *NDUFB2*, *BRAF*, *RNU685P*, *CCT4P1*, *MRPS33*, *TMEM178B*, *NDUFB10BP2*, *AGK*,*DENND11*, *WEE2*, *SSBP1*, *TAS2R3*, *TAS2R4*, *TAS2R6P*, *TAS2R5*, *MTND1P3*, *MYL6P4*, *PRSS37*, OR9A3P, *OP9A1P*, *PRSS3P3*, *TRBV1*, *TRBV2*, *TRBV3‐1*, *TRBV4‐1*, *TRBV5‐1*, *TRBV6‐1*, *TRBV7‐1*, *TRBV4‐2*, *TRBV6‐2*, *TRBV7‐2*, *TRBV8‐1*, *TRBV5‐2*, *TRBV6‐4*, *TRBV7‐3*, *TRBV8‐2*, *TRBV5‐3*, *TRBV9*, *TRBV10‐1*, *TRBV11‐1*, *TRBV12‐1*, *TRBV12‐2*, *TRBV6‐5*, *TRBV6‐8*, *TRBV7‐7*, *TRBV5‐7*, *TRBV7‐9*, *TRBV13*, *TRBV10‐3*, *TRBV11‐3*, *TRBV12‐3*, *TRBV12‐4*, *TRBV12‐5*, T*RBV14*, *TRBV15*, *TRBV16*, *TRBV17*, *TRBV18*, *TRBV19*, *TRBV20*, *TRBV21‐1*, *TRBV22‐1*, *TRBV23‐1*, *TRBV24‐1*, *TRBV25‐1*, *TRBVA*, *TRBV26*, *TRBVB*, *TRBV27*, *TRBV28*, *PGBD4‐1*, *TBV29‐1*, *PRSS1*, *PRSS2*, *PRSS3‐1*, *WBP1LP1*, *TRBD1*, *TRBJ1‐1*, *TRBV1‐2*, *TRBG1‐3*, *TRBJ1‐4*, *TRBJ1‐5*, *TRBJ1‐6*, *TRBC1*, *TRBJ2‐1*, *TRBJ2‐2*, *TRBJ2‐2P*, *TRBJ2‐3*, *TRBJ2‐4*, *TRBJ2‐5*, *TRBJ2‐6*, *TRBJ2‐7*, *TRBC2*, *TRBV30*, *EPHB6*, *TRPV6*, *TRPV5*, *LLCFC1*, *KEL*,*OR9A2*, *OR9P1P*, *OR6V1*, *OR6W1P*, *PIP*,*TAS2R39*, *TAS2R40*, *GSTK1*, *TMEM139*, *CASP2*, *RN7SL535P*, *RN7SL481*, *PMHINT1P1*, *CLCN1*, *FAM131B*, *ZYX*, *MIR6892*, *EPHA1*, *TAS2R62P*, *TAS2R60*, *TAS2R41*, *OR2R1P1*, *OR10AC1*, *PAICSP5*, *CTAGE15*, *RNU6‐162P*, *TCAF1P1*, *TCAF2*, *TCAF2C*

*Note:* Results obtained using the processed HDR‐del method.

## 4 Discussion

We were able to diagnose Sengers syndrome by a comprehensive genomic analysis. We identified both large chromosomal deletions containing the *AGK* gene which is inherited from the asymptomatic mother and a pathogenic *AGK* variant which is inherited from the asymptomatic father. The combination of RNA‐seq and WGS revealed the potential presence of a large deletion. However, RNA‐seq is not always possible, in part due to limited sample availability. In such cases, comparisons of results obtained from multiple bioinformatics tools using WGS data could be useful for identifying large chromosomal deletions within segmental duplication regions. Several bioinformatics tools (Abyzov et al. 2011; Quinodoz et al. 2021; Zhu et al. 2012; Chen et al. 2016; Layer et al. 2014) estimate copy‐number variation using depth of coverage. Unlike these tools, the HDR‐del method used in this study compares genotype patterns in specific regions between two individuals using the HDR. By assigning genotypes to two categories, our HDR‐del approach has a relatively fast calculation time (Imai‐Okazaki et al. 2017). More specifically, the HDR‐del approach accounts for the fact that hemizygous chromosomal deletion regions lack heterozygous variants, resulting in long runs of homozygosity (ROH) in Variant Call Format (VCF) files generated from WGS. In the HDR‐del approach, we calculate the “difference” in heterozygous status between an affected individual and control individuals and controls (Imai et al. 2016) over all candidate chromosomal deletion regions, defined as ROH longer than 1 Mbp with appropriate test statistic.

In our case with Sengers syndrome, we demonstrate that different types of bioinformatics approaches, one based on coverage information and another based on Hamming distance, could identify candidate chromosomal deletions in complicated regions, such as segmental duplications. Previous study reported that the ATAD3 region is the only known gene causative for mitochondrial disease where segmental duplications lead to recurrent non‐allelic homologous recombination (Frazier et al. 2021). Our result suggests the possibility that other genes located in segmental duplication regions may cause mitochondrial diseases.

In conclusion, we successfully diagnosed Sengers syndrome by detecting a known pathogenic variant and newly reported large chromosomal deletion within a segmental duplication by a comprehensive genomic analysis, including RNA‐seq, WGS, and panel sequencing. Our results also highlight the importance of considering segmental duplications when inconsistent results for candidate deletion regions are obtained from different bioinformatics pipelines. To identify chromosomal deletions in segmental duplications, optimizing parameters for bioinformatics tools could be useful.

## Author Contributions

K.N., A.I., and Y.Y. wrote the manuscript. S.N., Y.S., T.O., T.E., T.T., T.F., A.O., and K.M. provided the clinical information. A.H., T.K., and A.K. analyzed the data. Y.Y. and Y.K. performed the experiments. All authors discussed the results and commented on the manuscript.

## Conflicts of Interest

The authors declare no conflicts of interest.

## Supporting information


Data S1.

## Data Availability

The data sets generated and/or analyzed in the current study are available from the corresponding author upon reasonable request.
