# Supplementary material for: Successful Diagnosis of Sengers Syndrome Using a Comprehensive Genomic Analysis
Source: Mol Genet Genomic Med. 2025 Jan 16;13(1):e70048. doi: 10.1002/mgg3.70048 (PMC11736639; doi:10.1002/mgg3.70048)
Supplement: Supplementary file 1 — Data S1. [file MGG3-13-e70048-s001.docx]

**Supplementary Data S1**

**Cardiac echocardiography**

**
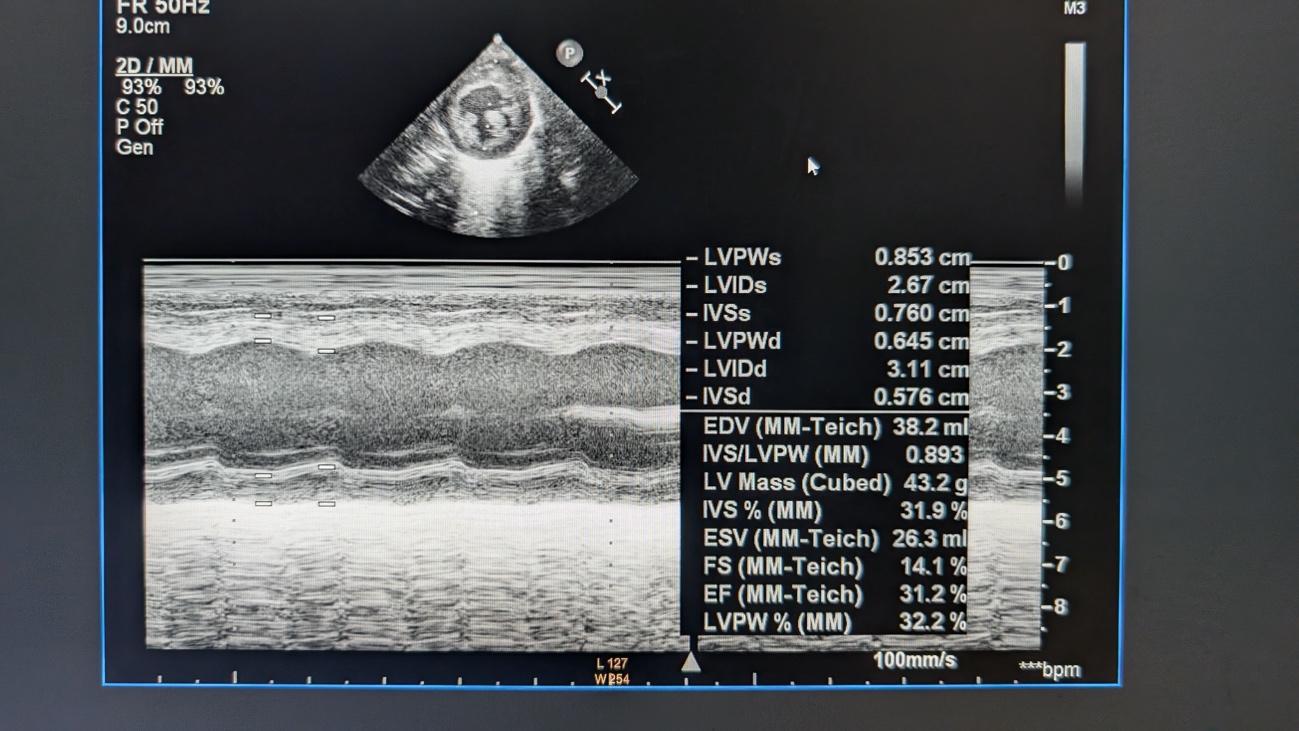
**

**Supplementary Data S2**

**Panel sequencing**

Total DNA was extracted for a genetic analysis from blood samples of the patient and his parents using standard protocols. Panel sequencing was performed with a panel of 367 genes involved in mitochondrial disease and whole mitochondrial DNA using the IDT platform (Kanagawa, Japan), as described previously(1).

**Whole genome sequencing**

Whole genome sequencing (WGS) was performed to screen for large chromosomal deletions. Briefly, 150 bp paired-end reads were obtained using DNBSEQ (MGI Shenzhen, China) from a patient blood sample. The detailed protocol for WGS was described previously(2). WGS libraries were prepared from 100 ng of genomic DNA using an MGIEasy FS DNA Library Prep Kit v2.1 (MGItech, China) according to the manufacturer's instructions. Paired-end 150-bp sequencing was performed on a DNBSEQ-T7 sequencer (MGItech) using a DNBSEQ-T7 High Throughput Sequencing set (PE150) v1.0 (MGItech).

**RNA sequencing**

RNA sequencing (RNA-seq) was performed using DNBSEQ (MGI) to assess changes in gene expression in skin fibroblasts obtained from the patient. The protocol for RNA-seq has been described previously(2).

**Bioinformatics analysis**

Quality check of the raw sequence reads was done using FASTQC. Read trimming by base quality was performed using Trimmomatic (3). After removing of low-quality reads and adaptors, the clean reads were mapped to the human reference genome (GRCh38) using the Burrows-Wheeler Aligner (BWA) v0.7.17, the GRCh38 reference genome , Picard, and SAMtools.GATK v4.1.9 was used for insertion and deletion realignment, quality recalibration, and variant calling(4, 5). Detected variants were annotated using both ANNOVAR (version Mon, 16 Apr 2018) and custom Ruby scripts. BAM files obtained from WGS were used to screen deletion regions using CNVnator, ERDS, AutoMap, Manta, and LUMPY to detect structural abnormalities(6-10). OUTRIDER was used to analyze expression based on RNA-seq data(11). OUTRIDER uses a distribution of outlier counts per sample with an FDR of less than 0.05. Hamming distance ratio (HDR)-del, a unique statistical genetics method for screening chromosomal deletions using Hamming distance, was also used(12, 13).

In this method, homozygous stretches on each chromosome are screened and candidate chromosome deletions are prioritized based on a test statistic for the Hamming distance ratio (HDR) between case–case and case–control groups.

**Mitochondrial DNA Copy Number Analysis**

We measured the ratio of mitochondrial DNA copy numbers to reference nuclear DNA copy numbers using Bio-rad droplet digital PCR (California, USA) as described previously (14).

**Supplementary Data S3**

| % | ComplexⅠ | ComplexⅡ | ComplexⅢ | ComplexⅣ | Citrate  synthase |
| --- | --- | --- | --- | --- | --- |
| % of normal | 65 | 68 | 154 | 177 | 122 |
| CS ratio | 51 | 54 | 123 | 140 |  |
| Co Ⅱ ratio | 95 |  | 219 | 225 |  |

Supplementary Data S3: The results of the mitochondrial respiratory chain (MRC) activity analysis performed on this patient's liver tissue revealed a reduction in complex I enzyme activity. CS: Citrate Synthase, Co: Complex

**Supplementary Data S4**

| P-value | Genome | annotation |
| --- | --- | --- |
| 0.04 | chr7:140,577,406  –142,453,641 | *RAB19*,*MKRN1*,*DENND2A*,*Y_RNA*,*RN7SL771P*,*ADCK2*,  *NDUFB2*,*BRAF*,*RNU685P*,*CCT4P1*,*MRPS33*,*TMEM178B*,  *NDUFB10BP2*,*AGK*,*DENND11*,*WEE2*,*SSBP1*,*TAS2R3*,*TAS2R4*,TAS2R6P,*TAS2R5*,*MTND1P3*,*MYL6P4*, *PRSS37*,*OR9A3P*, *OP9A1P*, *PRSS37*,*OR9A3P*,*OP9A1P*,*PRSS3P3, TRBV1*,  *TRBV2*,*TRBV3-1*,*TRBV4-1*,*TRBV5-1*,*TRBV6-1*,*TRBV7-1*,*TRBV4-2*,*TRBV6-2*,*TRBV7-2*,*TRBV8-1*,*TRBV5-2*,*TRBV6-4*,*TRBV7-3*,*TRBV8-2*,*TRBV5-3*,*TRBV9*,*TRBV10-1*,*TRBV11-1*,*TRBV12-1*,*TRBV12-2,TRBV6-5* |
| 0.04 | chr7:142,501,509  –143,742,039 | *TRBV6-8*,*TRBV7-7*,*TRBV5-7*,*TRBV7-9*,*TRBV13*,*TRBV10-3*,  *TRBV11-3*,*TRBV12-3*,*TRBV12-4*,*TRBV12-5*,*TRBV14*,*TRBV15*,  *TRBV16*,*TRBV17*,*TRBV18*,*TRBV19*,*TRBV20*,*TRBV21-1*,*TRB*  *V22-1*,*TRBV23-1*,*TRBV24-1*,*TRBV25-1*,*TRBVA*,*TRBV26*,*TRB*  *VB*,*TRBV27*,*TRBV28*,*PGBD4-1*,*TRBV29-1*,*PRSS1*,*PRSS2*,  *PRSS3-1*,*WBP1LP1*,*TRBD1*,*TRBJ1-1*,*TRBJ1-2*,*TRBG1-3*,*TR*  *BJ1-4*,*TRBJ1-5*,*TRBJ1-6*,*TRBC1*,*TRBJ2-1*,*TRBJ2-2*,*TRBJ2-2P*,*TRBJ2-3*,*TRBJ2-4*,*TRBJ2-5*,*TRBJ2-6*,*TRBJ2-7*,*TRBC2*,*TR*  *BV30*,*EPHB6*,*TRPV6*,*TRPV5*,*LLCFC1*,*KEL*,*OR9A2*,*OR9P1P*,  *OR6V1*,*OR6W1P*,*PIP*,*TAS2R39*,*TAS2R40*,*GSTK1*,*TMEM139*,  *CASP2*,*RN7SL535P*,*RN7SL481P*,*HINT1P1*,*CLCN1*,*FAM131B*,  *ZYX*,*MIR6892*,*EPHA1*,*TAS2R62P*,*TAS2R60*,*TAS2R41*,*OR2R1*  *P*,*OR10AC1*,*PAICSP5*,*CTAGE15*,*RNU6-162P*,*TCAF1P1*,*TCA*  *F2*,*TCAF2C* |

Supplementary Data S4: Unprocessed HDR-del results

Unprocessed HDR-del results. Genome shows the candidate regions. Annotation indicates genes included in the regions. Using the unprocessed HDR-del method, a 4.7 kb region was not detected due to segmental duplications.

**Supplementary Data S5**


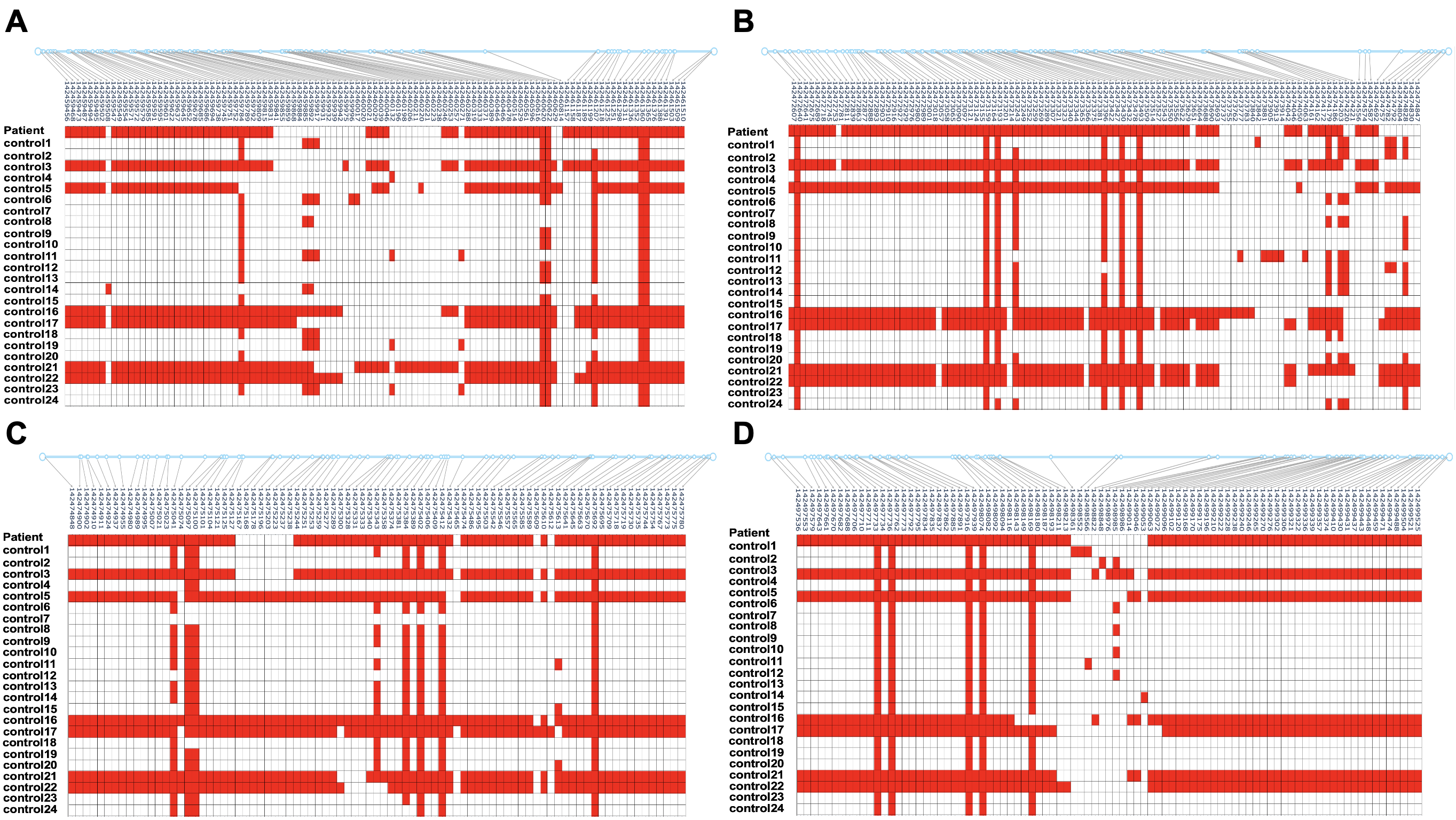


Supplementary Materials S5: Visualization of regions not detected by HDR-del. Heterozygous variants are highlighted in red, wild-type and homozygous variants are highlighted in white.

HDR-del contained many heterozygous variants not only in the patients but also in six controls in this group. Vertical line is sample name. Horizontal line is variant loci.

A: Genotypes of the patient and control group were visualized in the region 142,459,456-142,461,510 were visualized. We observed a large number of heterozygous variants in the patient and 6 controls. B: Genotypes of the patient and control group in the region 142,472,607-142,474,847 were visualized. C: Genotypes of the patient and controls in the region 142,474,848-142,499,525 were visualized. D: Genotypes of the patient and control group in the region 142,497,536-142,499,525 were visualized

**Supplementary Data S6**


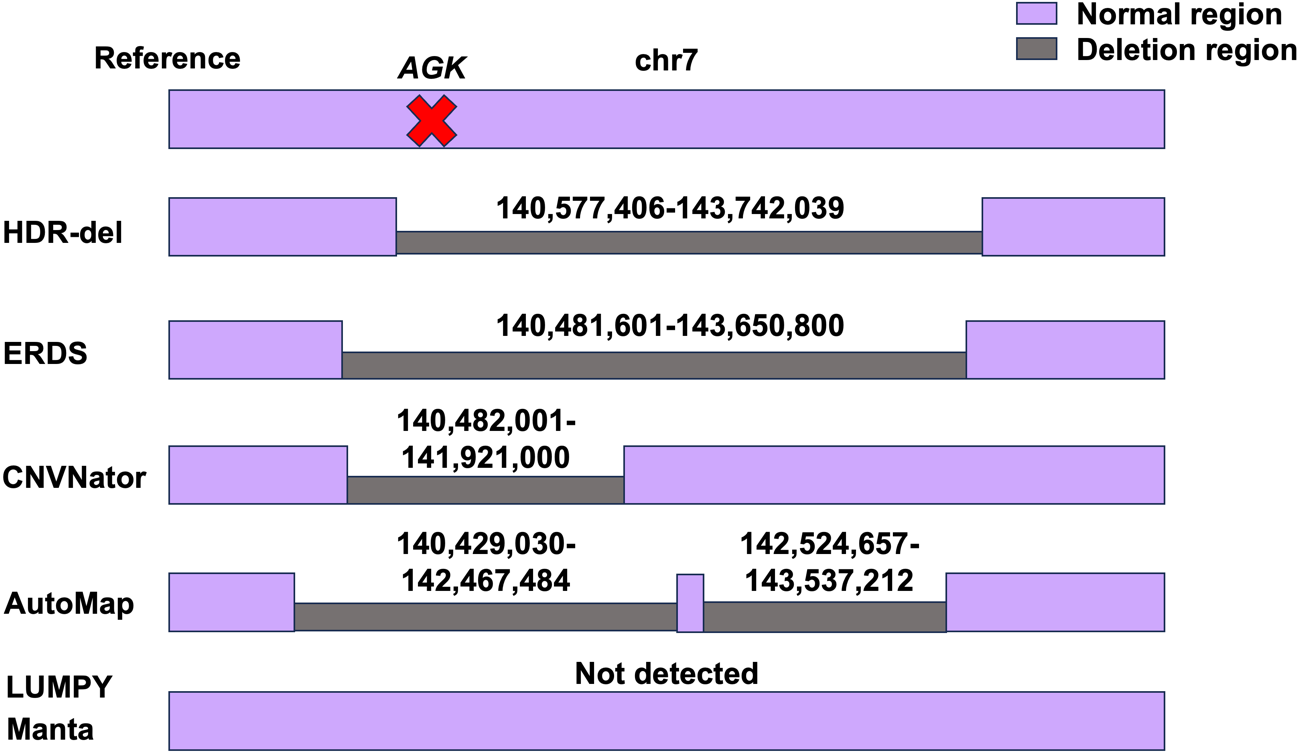


Supplementary Materials S6: Comparison of deletion regions detected by our HDR-del method with existing bioinformatics tools for structural aberration detection.

**Reference**

1. Naganuma T, Imasawa T, Nukui I, Wakasugi M, Kitamura H, Yatsuka Y, et al. Focal segmental glomerulosclerosis with a mutation in the mitochondrially encoded NADH dehydrogenase 5 gene: A case report. Mol Genet Metab Rep. 2023;35:100963.

2. Omichi N, Kishita Y, Nakama M, Sasai H, Terazawa A, Kobayashi E, et al. Novel ITPA variants identified by whole genome sequencing and RNA sequencing. J Hum Genet. 2023;68(9):649-52.

3. Bolger AM, Lohse M, Usadel B. Trimmomatic: a flexible trimmer for Illumina sequence data. Bioinformatics. 2014;30(15):2114-20.

4. Li H, Durbin R. Fast and accurate short read alignment with Burrows-Wheeler transform. Bioinformatics. 2009;25(14):1754-60.

5. McKenna A, Hanna M, Banks E, Sivachenko A, Cibulskis K, Kernytsky A, et al. The Genome Analysis Toolkit: a MapReduce framework for analyzing next-generation DNA sequencing data. Genome Res. 2010;20(9):1297-303.

6. Zhu M, Need AC, Han Y, Ge D, Maia JM, Zhu Q, et al. Using ERDS to infer copy-number variants in high-coverage genomes. Am J Hum Genet. 2012;91(3):408-21.

7. Abyzov A, Urban AE, Snyder M, Gerstein M. CNVnator: an approach to discover, genotype, and characterize typical and atypical CNVs from family and population genome sequencing. Genome Res. 2011;21(6):974-84.

8. Quinodoz M, Peter VG, Bedoni N, Royer Bertrand B, Cisarova K, Salmaninejad A, et al. AutoMap is a high performance homozygosity mapping tool using next-generation sequencing data. Nat Commun. 2021;12(1):518.

9. Chen X, Schulz-Trieglaff O, Shaw R, Barnes B, Schlesinger F, Kallberg M, et al. Manta: rapid detection of structural variants and indels for germline and cancer sequencing applications. Bioinformatics. 2016;32(8):1220-2.

10. Layer RM, Chiang C, Quinlan AR, Hall IM. LUMPY: a probabilistic framework for structural variant discovery. Genome Biol. 2014;15(6):R84.

11. Brechtmann F, Mertes C, Matuseviciute A, Yepez VA, Avsec Z, Herzog M, et al. OUTRIDER: A Statistical Method for Detecting Aberrantly Expressed Genes in RNA Sequencing Data. Am J Hum Genet. 2018;103(6):907-17.

12. Imai-Okazaki A, Kohda M, Kobayashi K, Hirata T, Sakata Y, Murayama K, et al. HDR-del: A tool based on Hamming distance for prioritizing pathogenic chromosomal deletions in exome sequencing. Hum Mutat. 2017;38(12):1796-800.

13. Imai-Okazaki A, Matsunaga A, Yatsuka Y, Nitta KR, Kishita Y, Sugiura A, et al. Long-term prognosis and genetic background of cardiomyopathy in 223 pediatric mitochondrial disease patients. Int J Cardiol. 2021;341:48-55.

14. Shoop WK, Gorsuch CL, Bacman SR, Moraes CT. Precise and simultaneous quantification of mitochondrial DNA heteroplasmy and copy number by digital PCR. J Biol Chem. 2022;298(11):102574.
